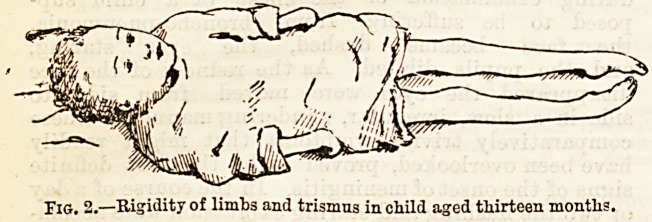# Spasm in the Diagnosis of Acute Meningitis in Children

**Published:** 1894-01-06

**Authors:** Theodore Fisher

**Affiliations:** Registrar to the Bristol General Hospital, late Registrar to the Royal Hospital for Children and Women, London, S.E.


### MEDICAL PROGRESS AND HOSPITAL CLINICS.

[*The Editor will be glad to receive offers of co-operation and contributions from members of the profession. All letters should be addressed to* THE EDITOR, THE LODGE, PORCHESTER SQUARE, LONDON, W.]

### SPASM IN THE DIAGNOSIS OF ACUTE MENINGITIS IN CHILDREN.

By THEODORE FISHER, M.D.Lond., Registrar to the Bristol General Hospital, late Registrar to the Royal Hospital for Children and Women, London, S.E.

The doctor's calling has been called the science and art of medicine. The practical man looks upon the art as the only division that is worth serious attention, but it must not be forgotten that it is the science that places its companion upon a firm foundation.

Yet all that is of immediate practical value is not confined to various methods of treatment; the importance of diagnosis and prognosis cannot be overestimated. The science may show that the art will be unavailing, and teach that some diseases, such as the one that we propose briefly to consider, are, unfortunately, beyond the reach of treatment. It does not follow, however, that such diseases must consequently be ignored. They are the practitioner's worst enemies, and, if only for the sake of his own reputation, he must be ever upon the alert to recognise their presence. To tell the parents of a sick child that an ailment is trivial, and on calling a few days later to find the patient evidently beyond all hope of recovery, is neither satisfactory to the friends nor likely to increase confidence in the medical attendant.

The difficulty of an early recognition of meningitis is well known, and the knowledge of that difficulty should stimulate to care and watchfulness in all cases where the least suspicion can be entertained.

A recent work on diseases of children describes the early symptoms as headache, vomiting, and hyperæsthesia. Such symptoms taken by themselves are of little value. The two first may be present in an ordinary dyspeptic attack, at the commencement of an exanthema, of typhoid fever, or of pneumonia, and personal experience must have told many of us that hyperæsthesia of various parts of the skin, and especially of the scalp, may be associated with febricula due to ordinary catarrh. In children who are suffering from some form of tubercular disease, more notice would naturally be taken of vomiting than in those in whom there is no suspicion of any such affection. If, for example, a boy confined to bed for disease of the hip or knee loses his natural cheerfulness and commences to vomit, further development is, perhaps, expected; or if a child suffering from some form of chest complaint, phthisis, empyema, or what not may have been diagnosed as broncho-pneumonia, becomes more lethargic and is attacked with vomiting, attention is at once directed to the brain.

Although headache and vomiting may suggest the gravity of the case, a definite diagnosis can rarely be made until some symptom pointing more directly to the brain presents itself. A transient squint may be noticed early by the watchful mother. About three years ago a friend spoke to me about his first baby, a fine healthy-looking boy, aged seven or eight months. The day before it had not seemed well, and an occasional squint was present. The family doctor was away and his *locum tenens* was called in, who said that the child would soon be perfectly well again. I promised to call, and the following day found unmistakable evidence of meningitis. In another four days the child was dead within a week from the appearance of the squint.

Twitchings are not uncommon manifestations of spasm of muscles of the face, which may occur early, but are more usual when the disease is advanced. The masseters also may occasionally become affected with spasm during the later stages of meningitis, but trismus may be the first symptom, as is shown by a case recently recorded by a French writer, M. Boix.

Commoner than any of the above is what may be described as the first stage of a fit. For example, during examination of the chest of a child supposed to be suffering from broncho-pneumonia, the face became flushed, the eyes staring, and the pupils dilated. As the redness of the face disappeared the eyes were moved from side to side in a slow, irregular, wandering manner. These comparatively trivial symptoms that might readily have been overlooked, proved to be the first definite signs of the onset of meningitis. In the course of a day or two the flushing and staring expression were accompanied by twitchings of the face and hurried respiration, and still later by rigidity of the limbs, followed

FIG. 1.—Retraction of neck and opisthotonos in child aged one and a-half years.

by tremor, until finally retraction of the neck and opisthotonos were ushered in.

This brings us to a form of spasm, retraction of the neck, that is rarely seen in any other condition than meningitis, although it is certainly not confined to that affection, nor by any means always present. It may be absent throughout the course of the disease, and usually does not make its appearance until there is little doubt about the diagnosis, yet like squint and spasm of the jaw it may occur earlier, or even be the first indication that anything is wrong. For example, a child aged eight months was brought up to the Royal Hospital for Children and Women for "rheumatism in the neck," the head having been "fastened back" for three weeks. Not until the day before had there been any vomiting, and even then the child did not appear very ill. Three weeks afterwards death occurred, and sero-purulent meningitis was found.

As the disease advances retraction of the neck may become further complicated by opisthotonos, both of which may be extreme.

A well-marked case as shown in Figure 1, in which, however, both the retraction of the neck and the opisthotonos became considerably worse before death took place. In a case such as this the spasm is most marked in the muscles at the back of the neck and in the back itself; in others, the back almost entirely escapes and the limbs are mainly affected. The spasm may take the form of uniform rigidity, or there may be frequently repeated exacerbations.

Either rigidity or varying spasm may be bilateral or limited to one side. When bilateral a not uncommon position of the forearms is that of marked pronation, associated with flexion of the wrists and some flexion of the fingers and thumbs.

Fig. 2.—Rigidity of limbs and trismus in child aged thirteen months.

In the case shown in Figure 2 there was extension of the legs with marked hyperextension of the feet, associated with some rigidity of the back and trismus, as well as spasmodic contraction of both forearms. Sudden increase of spasm occurred at frequent intervals. The case thus bore some resemblance to tetanus and to severe tetany. The condition of the hands, however, differed from that of both diseases. In tetanus they are usually unaffected, and in tetany there is lateral arching of the palm with the fingers in the interosseal position.

In the foregoing remarks very little else but spasm has been referred to, but it must not be forgotten that acute meningitis may run its course without any sign of spasm being present. Possibly paresis of a limb or of limbs may take its place, or paralysis of ocular or facial muscles. One other symptom may be mentioned, which is a manifestation neither of spasm nor of paralysis. In children in whom the anterior fontanelle is still patent, some idea can be obtained of the intracranial tension. This is of great value in distinguishing meningitis from cerebral symptoms associated with diarrhœa, or with some acute disease, such as pneumonia. In meningitis the fontanelle is tense, prominent, and frequently no pulsation is palpable, in diarrhœa, pulsation may also be slight, but the fontanelle is sunken, while in acute febrile diseases, although full and prominent, it is soft and pulsates markedly.

Of recent years considerable notice has been taken of an irregular pulse as an early symptom of meningitis. This is certainly of value, since although, an irregular pulse is not uncommon in children during convalescence from fevers, it rarely occurs at the onset. It may, however, follow a short attack of headache and vomiting, apparently due to ordinary dyspepsia.

## Figures and Tables

**Fig. 1. f1:**
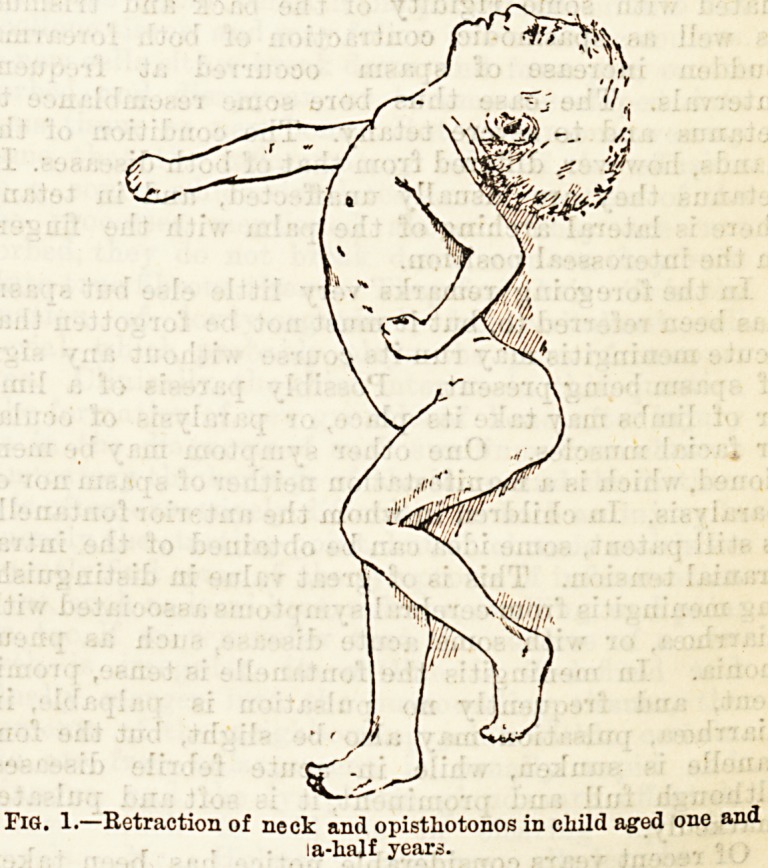


**Fig. 2. f2:**